# Antagonistic effects of smoking and maternal glycemia on fetal growth: a retrospective study among 13,958 pregnant French women

**DOI:** 10.3389/fendo.2025.1527358

**Published:** 2025-03-14

**Authors:** Emmanuel Cosson, Lionel Carbillon, Sopio Tatulashvili, Hélène Bihan, Eric Vicaut, Ines Barka, Sara Pinto, Imen Rezgani, Mohamed Zerguine, Jean-Jacques Portal, Marion Fermaut, Jardena J. Puder, Amélie Benbara

**Affiliations:** ^1^ Assistance Publique - Hôpitaux de Paris (AP-HP), Avicenne Hospital, Paris 13 University, Sorbonne Paris Cité, Department of Endocrinology-Diabetology-Nutrition, Centre de Recherche en Nutrition Humaine - Ile de France (CRNH-IdF), Centre Spécialisé de l’Obésite Île-de-France Nord, Bobigny, France; ^2^ Université Sorbonne Paris Nord and Université Paris Cité, Institut national de la santé et de la recherche médicale (INSERM), Institut national de recherche pour l’agriculture, l’alimentation et l’environnement (INRAE), Caisse Nationale Assurance Maladie (CNAM), Center of Research in Epidemiology and StatisticS (CRESS), Nutritional Epidemiology Research Team (EREN), Bobigny, France; ^3^ Assistance Publique - Hôpitaux de Paris (AP-HP), Jean Verdier Hospital, Paris 13 University, Sorbonne Paris Cité, Fédération Hospitalo-Universitaire “Early Identification of Individual trajectories in neuro-developmental disorders” (I2D2), Department of Perinatology and Gynecology, Bondy, France; ^4^ Assistance Publique - Hôpitaux de Paris (AP-HP), Unité de Recherche Clinique St-Louis-Lariboisière, Université Denis Diderot, Paris, France; ^5^ Obstetric Service, Department Woman-Mother-Child, Lausanne University Hospital, Lausanne, Switzerland

**Keywords:** birthweight, cigarettes, diabetes in pregnancy, gestational diabetes mellitus, hyperglycemia in pregnancy, pregnancy outcomes, smoking, tobacco

## Abstract

**Introduction:**

Smoking and hyperglycemia first diagnosed during pregnancy (H1inP) have opposing effects on fetal growth. The aim of this study was to explore adverse pregnancy outcomes, particularly fetal growth, according to the smoking and H1inP status.

**Methods:**

We included 13,958 women from a large French dataset (2012–2018). Using multivariable regression analyses, we retrospectively evaluated the risk of large-for-gestational-age (LGA) babies and other adverse outcomes according to the H1inP and smoking status in four groups: no H1inP/non-smoker (group A: *n* = 10,454, 88.2%), no H1inP/smoker (group B: *n* = 819, 5.9%), H1inP/non-smoker (group C: *n* = 2,570, 18.4%), and H1inP/smoker (group D: *n* = 115, 0.8%).

**Results:**

The rates of LGA were 8.9%, 4.0%, 14.6%, and 8.7% in groups A, B, C, and D, respectively (global ANOVA *p* < 0.0001, factor H1inP *p* = 0.0003, factor smoking *p* = 0.0002, and interaction *p* = 0.48). After adjustment for potential confounders including age, body mass index, employment, ethnicity, parity, hypertension before pregnancy, gestational weight gain, and alcohol and drug consumption, H1inP was associated with a higher risk [odds ratio (OR) = 1.50, 95% confidence interval (95%CI) = 1.30–1.74] and smoking with a lower risk (OR = 0.35, 95%CI = 0.25–0.50) of LGA. In addition, H1inP was associated with a lower total gestational weight gain and a lower rate of small-for-gestational-age (SGA) babies, but higher rates of hypertensive disorders and more frequent caesarean sections and admissions in the neonatal intensive care unit. Smoking was associated with higher rates of SGA, including severe SGA (<3rd centile), and this despite a higher total gestational weight gain. Smoking increased the risk of hypertensive disorders only in women with H1inP.

**Discussion:**

Smoking among women with H1inP could mask the risk of maternal hyperglycemia for LGA babies. This could provide a false sense of security for women with H1inP who smoke, particularly when assessing for LGA alone, but these women still face other risks to their health, such as hypertensive disorders and the health of the fetus.

## Introduction

Tobacco use is the main preventable cause of adverse perinatal outcomes, including fetal restriction and small-for-gestational-age (SGA) babies, preterm birth, congenital malformations, and fetal loss (1). These complications are likely driven by placental dysfunction through nicotine and toxin exposure, hypoxia, oxidative stress, and epigenetic modifications (1–4).

Hyperglycemia first detected in pregnancy (H1inP) represents one of the most frequent pregnancy complications (5–8). Despite care, H1inP remains associated with several adverse neonatal and maternal outcomes (5, 6, 9). One of the main adverse outcomes is having large-for-gestational-age (LGA) babies, which in turn increases the risk of shoulder dystocia, fetal distress, and the need for urgent caesarean delivery. Fetal overgrowth during H1inP is mainly related to uncontrolled high glucose levels (5–8). Preterm delivery, neonatal hypoglycemia, and higher rates of admissions in the neonatal intensive care unit (NICU), as well as higher rates of maternal hypertensive disorders, could also reflect a poor glycemic control in the context of H1inP (5–9).

Despite careful prenatal management and smoking cessation assistance, a significant number of pregnant women with H1inP continue to smoke tobacco (10). In these women, we hypothesized that smoking and H1inP could have i) opposing effects on fetal growth, but ii) distinct and even synergistic combined effects on other adverse perinatal outcomes. Indeed, a normal fetal growth in women with H1inP who smoke could falsely reassure caregivers about the impact of glucose control and the risk of other H1inP-related adverse outcomes. Reciprocally, a normal fetal growth in smokers due to H1inP could mask fetal growth restriction and placental dysfunction. In this context, we explored these outcomes in a large French dataset according to the smoking and H1inP status.

## Materials and methods

### Our cohort

This observational cohort study was conducted at the Jean Verdier University Hospital in Bondy, a suburb of Paris, France. According to French law (31/07/1991, *programme de médicalisation des systèmes d’information*), healthcare establishments shall carry out a medical assessment and analysis of their activities. Thus, perinatal data are routinely and prospectively registered at birth for all women giving birth at the university hospital by the midwife assisting the delivery, and then checked and collected during the maternity stay by a midwife qualified in data management and storage. At our perinatal center, all patients are informed during their first prenatal visit that their medical records may be used for the assessment and improvement of our procedures, unless they oppose. Analyses were based on data from the hospital’s routine electronic medical records of outcomes during pregnancy and at birth, which occurred between January 2012 and December 2018 (11–16). All data were analyzed anonymously. Our database is registered in the French Committee for computerized data (Commission Nationale de l’Informatique et des Libertés, no. 1704392v0).

### Selection criteria for the present study sample

**Figure 1:**
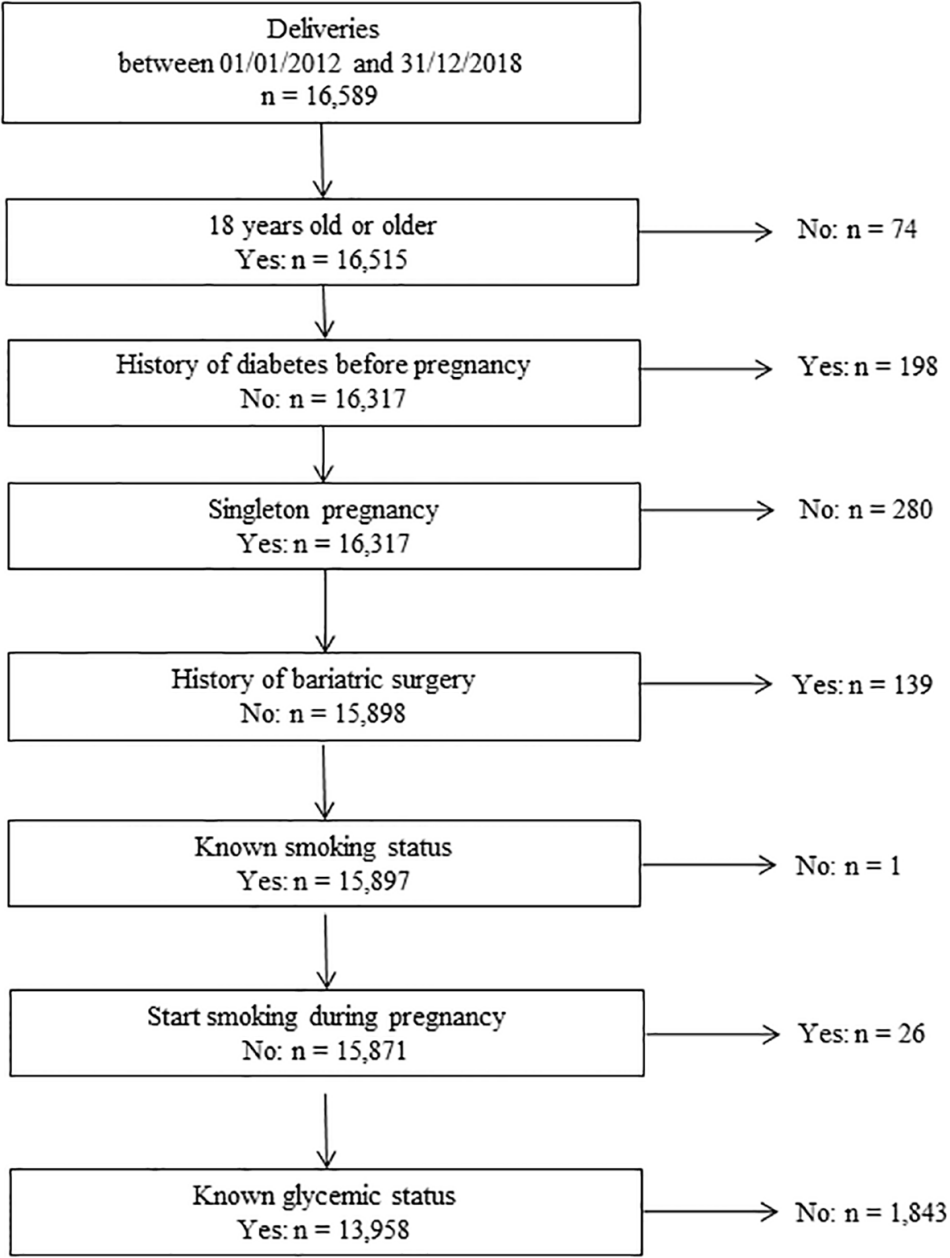
Flowchart of the study.

The inclusion criteria for the women comprising the present study sample were as follows: delivery between January 2012 and December 2018; age of at least 18 years; no known diabetes before pregnancy; a single fetus pregnancy; no history of bariatric surgery; a known smoking status at the beginning of prenatal care, with the exclusion of women having begun to smoke during pregnancy; and a known H1inP status (**Figure 1**).

### H1inP screening and care

The French recommendations for H1inP screening, diagnostic criteria, and care (6) were followed, except that universal screening was preferred over selective screening given the high prevalence of risk factors in our hospital population (14). Screening was performed at the beginning of pregnancy and between 24 and 28 weeks of gestation (WG) if initial screening was not performed or provided a normal result. Early screening was based on a fasting plasma glucose (FPG) measurement. Women with a FPG level ≥5.1 mmol/L were promptly provided care for H1inP. Women not diagnosed early with H1inP underwent an oral glucose tolerance test (OGTT) between 24 and 28 WG, where the FPG and the plasma glucose 1 h (1h-PG) and 2 h after OGTT (2h-PG) were measured (12). The International Association of Diabetes Pregnancy Study Group/World Health Organization (5, 7) recommendations were used to diagnose H1inP in accordance with the French regulations. H1inP was defined as a FPG ≥5.1 mmol/L and/or 1h-PG ≥10.0 mmol/L and/or 2h-PG ≥8.5 mmol/L (17).

All women diagnosed with H1inP were referred to our multidisciplinary team, which comprises a diabetologist, an obstetrician, a midwife, a dietician, and a diabetes nurse educator. Care was provided in accordance with the French recommendations. Specifically, our team provided individually tailored dietary advice and instructions to pregnant women on how to perform self-monitoring of their blood glucose levels six times a day (17). Women received insulin therapy when the pre-prandial and/or 2-h post-prandial capillary glucose levels were greater than 5.3 and 6.7 mmol/L, respectively, during follow-up. The obstetrical care provided also followed French recommendations (6).

### Data collection

Smoking status was self-reported and classified into two categories: “non-smokers” were those women who did not smoke at conception and those who ceased smoking because of the current pregnancy; “smokers” were those who continued smoking during pregnancy (10). The body mass index (BMI) was calculated according to the self-reported weight before pregnancy and the height measured during pregnancy. Ethnicity was self-reported as European, North African, Sub-Saharan African, Indian–Pakistani–Sri Lankan, Caribbean, or other. Data on the consumption of alcohol and recreation substances during pregnancy were self-reported.

### Outcomes

The following sets of outcomes were considered: termed “neonatal” and “maternal” perinatal outcomes by the INSPIRED research group (8). The primary outcome was LGA (>90th percentile) infant (18). The secondary neonatal outcomes included birth weight, SGA (<10th percentile) and severe SGA (<10th percentile) and babies (18), gestational age at birth and preterm delivery (any birth occurring after 22 WG and before 37 WG), and admissions in the NICU. The following exploratory outcomes (far less frequent than the former outcomes) were also considered: shoulder dystocia (defined as the use of obstetrical maneuvers: McRoberts episiotomy after delivery of the fetal head, suprapubic pressure, posterior arm rotation to an oblique angle, rotation of the infant by 180°C, and delivery of the posterior arm) (19); neonatal hypoglycemia (at least one blood glucose measurement under 2.5 mmol/L during the first 2 days of life); fetal or neonatal death (i.e., in the first 24 h of life) or stillbirth; and any birth malformations (11–16).

The secondary maternal outcomes included gestational weight gain (GWG; i.e., the weight measured before delivery minus the self-reported pre-pregnancy weight); insulin therapy for H1inP (as this is the only pharmacological therapy permitted in France); mode of birth, including induced delivery and unscheduled (before the scheduled date or during ongoing delivery) cesarean section; and hypertensive disorders (e.g., chronic hypertension, pregnancy-induced hypertension, and/or preeclampsia). The definitions of these events have been provided in previous publications (11–16).

### Statistical analyses

Continuous variables were expressed as mean ± standard deviation (SD). Categorical variables were expressed as frequencies and percentages. No data replacement procedure was used for missing data. ANOVA was used to compare continuous variables, while the chi-squared (*χ* ^2^) test or Fisher’s exact test was used as appropriate to compare categorical variables.

With regard to the characteristics of the included women (**Table 1**), the global difference between the four groups was first examined using a global one-way ANOVA; if a significant difference was found, a two-factor ANOVA was used to analyze more specifically potential differences related to the factors H1inP status (factor H1inP), smoking status (factor smoking), and their interaction (H1inP–smoking interaction).

**Table 1:** Patient characteristics according to the glycemic and smoking status.

	Total (*N* = 13,958)	No H1inP		H1inP		Factor H1inP	Factor smoking	Interaction	Global ANOVA
		Non-smoker: group A (*n* = 10,454)	Smoker: group B (*n* = 819)	Non-smoker: group C (*n* = 2,570)	Smoker: group D (*n* = 115)	*p*-value	*p*-value	*p*-value	*p*-value
Characteristics of the women									
Age (years)	30.5 ± 5.6	30.2 ± 5.5	28.7 ± 5.9	32.4 ± 5.4	32.7 ± 5.8	**<0.00001**	**0.0305**	**0.0017**	**<0.00001**
Pre-pregnancy body mass index (kg/m^2^)	25.1 ± 5.0	24.7 ± 4.8	23.3 ± 4.5	27.1 ± 5.5	26.7 ± 5.3	**<0.00001**	**0.0004**	**0.0441**	**<0.00001**
Pre-pregnancy obesity	2,279 (16.9%)	1,463 (14.5%)	86 (10.7%)	700 (28.0%)	30 (26.8%)	**<0.00001**	0.1038	0.2513	**<0.00001**
Family history of diabetes	3,689 (26.4%)	2,563 (24.5%)	228 (27.8%)	855 (33.3%)	43 (37.4%)	**<0.00001**	0.0980	0.9671	**<0.00001**
Employment at the beginning of pregnancy	5,322 (38.7%)	4,058 (39.4%)	361 (44.7%)	843 (33.5%)	60 (52.6%)	0.7622	**<0.00001**	**0.00052**	**<0.00001**
Hypertension before pregnancy	108 (0.8%)	62 (0.6%)	2 (0.2%)	41 (1.6%)	3 (2.6%)	**0.0003**	0.6794	0.1386	**<0.00001**
Parity	2.14 ± 1.25	2.12 ± 1.25	1.96 ± 1.09	2.31 ± 1.28	2.26 ± 1.51	**<0.00001**	0.1013	0.4066	**<0.00001**
Ethnicity						**0.0077**	**<.0001**	0.0528	**<0.00001**
Sub-Saharan African	2,818 (20.2%)	2,326 (22.3%)	46 (5.6%)	436 (17.0%)	10 (8.7%)				
North African	4,049 (29.1%)	2,976 (28.5%)	116 (14.2%)	927 (36.1%)	30 (26.1%)				
Caribbean	779 (5.6%)	636 (6.1%)	32 (3.9%)	108 (4.2%)	3 (2.6%)				
European	3,833 (27.5%)	2,762 (26.5%)	526 (64.5%)	484 (18.9%)	61 (53.0%)				
Indian–Pakistani–Sri Lankan	1,389 (10.0%)	955 (9.1%)	1 (0.1%)	433 (16.9%)	0 (0.0%)				
Other	1,068 (7.7%)	784 (7.5%)	94 (11.5%)	179 (7.0%)	11 (9.6%)				
Previous pregnancy(ies)									
History of H1inP						**<0.00001**	0.0869	0.4670	**<0.00001** ^a^
First child	5,283 (37.8%)	4,063 (38.9%)	362 (44.2%)	814 (31.7%)	44 (38.3%)				
No	7,924 (56.8%)	6,074 (58.1%)	443 (54.1%)	1,350 (52.5%)	57 (49.6%)				
Yes	751 (5.4%)	317 (3.0%)	14 (1.7%)	406 (15.8%)	14 (12.2%)				
History of macrosomia						**0.0031**	**0.0468**	0.5961	**<0.00001** ^a^
First child	5,283 (37.8%)	4,063 (38.9%)	362 (44.2%)	814 (31.7%)	44 (38.3%)				
No	8,241 (59.0%)	6,121 (58.6%)	448 (54.7%)	1,605 (62.5%)	67 (58.3%)				
Yes	434 (3.1%)	270 (2.6%)	9 (1.1%)	151 (5.9%)	4 (3.5%)				
History of renal vascular diseases in pregnancy						**0.0030**	0.5599	0.2731	**<0.00001** ^a^
First pregnancy	3,427 (24.6%)	2,740 (26.2%)	170 (20.8%)	499 (19.4%)	18 (15.7%)				
No	10,214 (73.2%)	7,506 (71.8%)	638 (77.9%)	1,978 (77.0%)	92 (80.0%)				
Yes	317 (2.3%)	208 (2.0%)	11 (1.3%)	93 (3.6%)	5 (4.3%)				
History of fetal death						**0.0336**	0.7430	0.3546	**0.0345** ^a^
First pregnancy	3427 (24.6%)	2,740 (26.2%)	170 (20.8%)	499 (19.4%)	18 (15.7%)				
No	10,225 (73.3%)	7,505 (71.8%)	634 (77.4%)	1,994 (77.6%)	92 (80.0%)				
Yes	306 (2.2%)	209 (2.0%)	15 (1.8%)	77 (3.0%)	5 (4.3%)				
History of fetal growth restriction						0.5905	0.0720	0.7719	**0.0173** ^a^
First pregnancy	3427 (24.6%)	2,740 (26.2%)	170 (20.8%)	499 (19.4%)	18 (15.7%)				
No	10,023 (71.8%)	7,352 (70.3%)	601 (73.4%)	1,979 (77.0%)	91 (79.1%)				
Yes	508 (3.6%)	362 (3.5%)	48 (5.9%)	92 (3.6%)	6 (5.2%)				
Habits during pregnancy									
Alcohol consumption	17 (0.1%)	8 (0.1%)	6 (0.7%)	1 (0.0%)	2 (1.7%)	0.8827	**<0.0001**	0.2478	**<0.00001**
Drug consumption	70 (0.5%)	28 (0.3%)	34 (4.2%)	2 (0.1%)	6 (5.2%)	0.2468	**<0.0001**	0.0864	**<0.00001**

Data are shown as *n* (percentage) or mean ± standard deviation. Data for the study sample (13,958 women) are available. p<0.05 are written in bold.

*H1inP*, hyperglycemia first diagnosed in pregnancy; *OGTT*, oral glucose tolerance test; *WG*, weeks of gestation.

a Yes *vs*. No (no history possible if first child)

**Figure 2:**
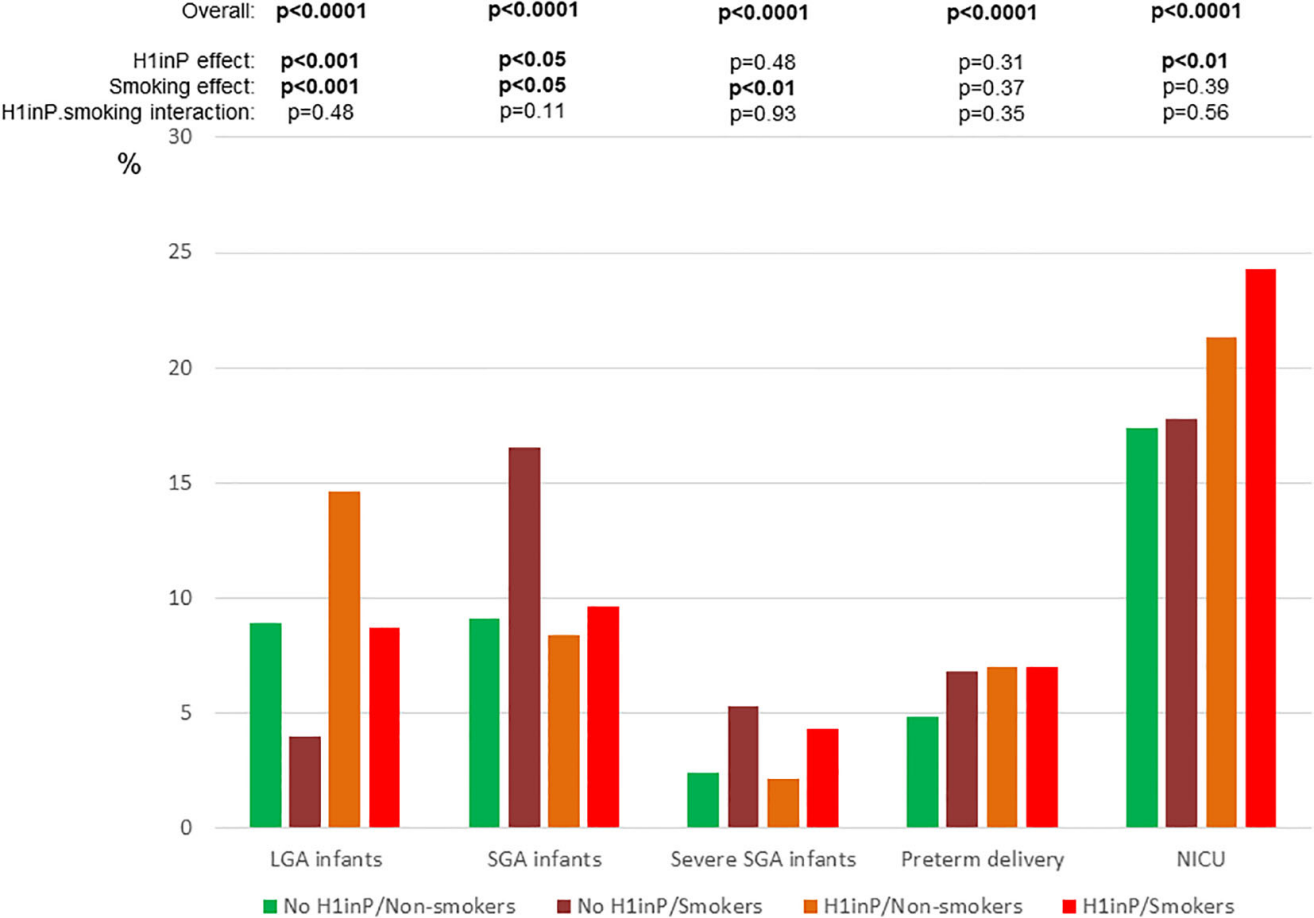
Primary and secondary neonatal outcomes according to glycemic and smoking status. *H1inP*, hyperglycemia first diagnosed in pregnancy; *LGA*, large-for-gestational-age; *NICU*, neonatal intensive care unit; *SGA*, small-for-gestational-age. p<0.05 are written in bold.

**Figure 3:**
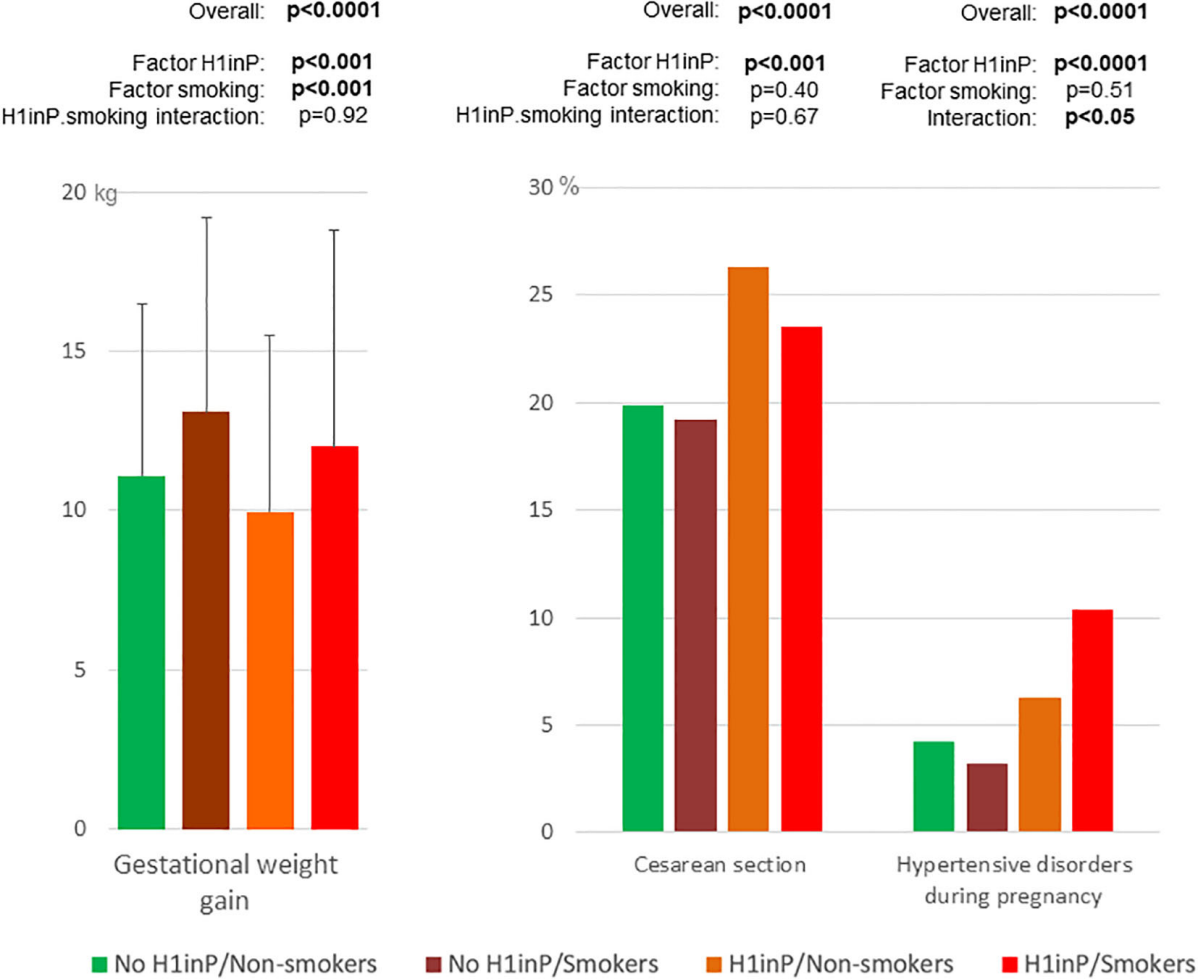
Secondary maternal outcomes according to glycemic and smoking status. *H1inP*, hyperglycemia first diagnosed in pregnancy. p < 0.05 are written in bold.

The rates of adverse pregnancy outcomes were compared according to the H1inP and smoking status (**Figures 2**, **3**, **Table 2**).

**Table 2:** Neonatal and maternal adverse pregnancy outcomes in the four groups of women categorized by the presence or absence of hyperglycemia first diagnosed in pregnancy and smoking.

	Available data	No H1inP		H1inP						
		Smoking No Group A	Smoking Yes Group B	Smoking No Group C	Smoking Yes Group D	Factor H1inP	Factor Smoking	Interaction	Global ANOVA	
		n=10,454	n=819	n=2,570	n=115		p	p	p	p
Neonatal outcomes										
**Primary**										
Large-for-gestational-age infant	n=13,958	935 (8.9%)	33 (4.0%)	374 (14.6%)	10 (8.7%)		**0.0003**	**0.0002**	0.4819	**<0.0001**
**Secondary**										
Birthweight (g)	n=13,958	3,296 ± 499	3,111 ± 507	3,344 ± 536	3,163 ± 581		0.0528	**<0.0001**	0.9483	**<0.0001**
Small-for-gestational-age infant	n=13,958	952 (9.1%)	135 (16.5%)	217 (8.4%)	11 (9.6%)		**0.0376**	**0.0165**	0.1115	**<0.0001**
Severe small-for-gestational-age infant		256 (2.4%)	43 (5.3%)	54 (2.1%)	5 (4.3%)		0.4837	**0.0023**	0.9348	**<0.0001**
Preterm delivery	n=13,958	507 (4.8%)	56 (6.8%)	180 (7.0%)	8 (7.0%)		0.3090	0.3740	0.3548	**<0.0001**
Neonatal intensive care unit	n=13,958	1,814 (17.4%)	146 (17.8%)	547 (21.3%)	28 (24.3%)		**0.0073**	0.3975	0.5611	**<0.0001**
**Exploratory**										
Shoulder dystocia	n=13,958	11 (0.1%)	2 (0.2%)	2 (0.1%)	0 (0.0%)					0.4379
Neonatal hypoglycemia	n=8,913	56 (0.9%)	5 (1.0%)	43 (2.0%)	1 (1.0%)		0.4660	0.5871	0.4929	**0.0017**
Neonatal death and stillbirth	n=13957	32 (0.3%)	3 (0.4%)	8 (0.3%)	1 (0.9%)					0.4983
Any malformation	n=13958	115 (1.1%)	7 (0.9%)	50 (1.9%)	4 (3.5%)		0.4691	0.3219	0.4861	**0.0010**
Maternal outcomes (secondary)										
Gestational weight gain (kg)	n=12,331	11.1 ± 5.4	13.1 ± 6.1	9.95 ± 5.55	12.0 ± 6.8		**<0.0001**	**<0.0001**	0.9292	**<0.0001**
Caesarean section	n=13,958	2,084 (19.9%)	157 (19.2%)	677 (26.3%)	27 (23.5%)		**0.0106**	0.4051	0.6660	**<0.0001**
Hypertensive disorders during pregnancy	n=13,958	442 (4.2%)	26 (3.2%)	163 (6.3%)	12 (10.4%)		**<0.0001**	0.5150	**0.0256**	**<0.0001**
Pregnancy-induced hypertension	n=13,958	228 (2.2%)	17 (2.1%)	91 (3.5%)	7 (6.1%)		**<0.001**	0.2781	0.1948	**<0.001**
Preeclampsia	n=13,958	217 (2.1%)	9 (1.1%)	73 (2.8%)	5 (4.3%)		**<0.01**	0.7267	0.0621	**<0.001**

Data are *n* (percentage) or mean (standard deviation).

*H1inP*, hyperglycemia first diagnosed in pregnancy.

The effects of H1inP and smoking on the primary outcome (i.e., LGA babies) were also explored using multivariable logistic regression analyses adjusted for the following confounders: age, employment, ethnicity, parity, pre-pregnancy BMI, and hypertension before pregnancy in model 1; the same variables as in model 1 + gestational weight gain in model 2; the same variables as in model 2 + alcohol and recreational substance consumption in model 3; and the same variables as in model 3 + history of macrosomic infant in model 4 (**Table 3**).

**Table 3:** H1inP and smoking effects for large-for-gestational-age infant in multivariable analyses.

		Unadjusted	Model 1	Model 2	Model 3	Model 4
		OR (95%CI), *p*	OR (95%CI), *p*	OR (95%CI), *p*	OR (95%CI), *p*	OR (95%CI), *p*
Large-for-gestational-age infant	H1inP effect	**1.747 (1.539–1.983), *p* < 0.001**	**1.389 (1.21–1.595), *p* < 0.001**	**1.501 (1.298–1.736), *p* < 0.001**	**1.502 (1.299–1.737), *p* < 0.001**	**1.406 (1.211–1.632), *p* < 0.001**
	Smoking effect	**0.452 (0.331–0.617), *p* < 0.001**	**0.429 (0.309–0.596), *p* < 0.001**	**0.359 (0.252–0.511), *p* < 0.001**	**0.352 (0.247–0.503), *p* < 0.001**	**0.361 (0.252–0.518), *p* < 0.001**

Model 1: adjusted for age, body mass index, employment, ethnicity, parity, and hypertension before pregnancy; Model 2: model 1 + adjusted for gestational weight gain; Model 3: model 2 + adjusted for alcohol and drug consumption; Model 4: model 3 + adjusted for history of macrosomic infant. p < 0.05 are written in bold.

*H1inP*, hyperglycemia first-diagnosed in pregnancy; *OR*, odds ratio; *95%CI*, 95% confidence interval.

All tests were two-sided. Analyses were conducted using SAS 9.4 software (SAS Institute Inc., Cary, NC, USA).

## Results

### Study population characteristics

As shown in the flowchart in **Figure 1**, 13,958 women were included, of whom 2,685 (19.2%) had H1inP and 934 (6.7%) were smokers. **Table 1** shows the characteristics of the study population in the four mutually exclusive groups: no H1inP/non-smoker (group A: *n* = 10,454, 88.2%), no H1inP/smoker (group B: *n* = 819, 5.9%), H1inP/non-smoker (group C: *n* = 2,570, 18.4%), and H1inP/smoker (group D: *n* = 115, 0.8%). Women with H1inP were less likely to smoke than those without H1inP (4.3% *vs*. 7.3%, *p* < 0.01). Globally, the characteristics differed between groups, such as the higher age and BMI in the case of H1inP and the lower age and BMI in smokers. There was an H1inP*smoking interaction for age and BMI. For example, age was lower in smokers than in non-smokers in women without H1inP, whereas the inverse was observed in women with H1inP.

The prevalence of smoking differed by ethnicity, with the following decreasing percentages: European, 15.3%; other, 9.8%; Caribbean, 4.5%; North African, 3.6%; and Sub-Saharan African, 0.2%; there was only one Indian–Pakistani–Sri Lankan woman who smoked (*p* < 0.0001). Smokers were more likely to consume alcohol and recreational substances during pregnancy compared with non-smokers (**Table 1**).

### Adverse perinatal outcomes

The rates of LGA babies were 8.9%, 4.0%, 14.6%, and 8.7% in groups A, B, C, and D, respectively (global ANOVA *p* < 0.0001, factor H1inP *p* = 0.0003, factor smoking *p* = 0.0002, and interaction *p* = 0.48) (**Figure 2**). After adjustment for confounders, H1inP was associated with a higher risk and smoking with a lower risk of LGA infant in all four models (**Table 3**).

**Figure 2** (neonatal outcomes) and **Figure 3** (maternal outcomes) show that all adverse perinatal outcomes differed by H1inP–smoking groups (number/percentages in **Table 2**). H1inP was associated with a lower rate of SGA babies, more frequent NICU admissions, lower maternal GWG, and a higher rate of caesarean section and of hypertensive disorders. Smoking was associated with more severe and non-severe SGA babies and a higher GWG. Finally, the rate of hypertensive disorders was the highest (over 10%) in the women who had H1inP and who were smokers (H1inP*smoking interaction *p* < 0.05).

In women with H1inP, the rate of insulin therapy was similar in non-smokers and smokers (36.7% *vs*. 37.4%, *p* = 0.68), with lower insulin doses at the end of the pregnancy in the non-smokers compared with the smokers (25 ± 24 *vs*. 37 ± 35 IU, *p* < 0.01).

**Table 2** also shows the results of the exploratory neonatal outcomes, with differences for neonatal hypoglycemia and any malformations according to the H1inP–smoking groups.

## Discussion

### Main results

In this multiethnic cohort, 6.7% of women were smokers during pregnancy. Smoking during pregnancy was associated with a reduced risk of LGA babies and H1inP with an increased risk of LGA babies, even after adjustment for confounders. Importantly, smoking was also associated with a higher GWG and, despite this, with higher rates of—especially severe—SGA babies. H1inP was associated with a lower GWG and a lower rate of SGA babies. In total, the prevalence rates of LGA and SGA babies in smokers with H1inP were similar to those in non-smokers without H1inP. Thus, the presence of H1inP and smoking might mask the respective impact and interfere with the ability to use fetal growth as a reliable marker of glycemic overload or placental dysfunction. H1inP was associated with higher rates of hypertensive disorders and of caesarean sections and more frequent admissions in the NICU. The combination of smoking and H1inP was associated with the highest risk of hypertensive disorders and NICU admissions.

### Fetal growth, GWG, treatment, and complications of delivery

In this study, the birth weight and LGA rates were lower in smokers than in non-smokers, similar to that in another study (1), and were higher in women with than in those without H1inP, as previously reported (5, 6, 9). These differences remained after adjustment for confounders, including for differences in the BMI and GWG. In women with H1inP, smokers had a lower BMI compared with non-smokers, as shown in a previous study (20), but not in another cohort (20, 21). This was not found in the women with H1inP, probably due to older age and obesity being classical risk factors for H1inP (14).

The higher rate of LGA babies in women with H1inP indicates that, despite the lower GWG, current glycemic reduction is either too late or insufficient, although this was in accordance with the current guidelines regarding H1inP care (17). Thanks to our interdisciplinary care including the integration of dieticians, women with H1inP achieved lower GWG than those without. It should be noted that the women with H1inP in this cohort had a similar need for insulin treatment in both smokers and non-smokers. This contrasts with another study that found a higher rate of insulin therapy in smokers (21). However, the insulin dosages at the end of pregnancy were higher in smokers than in non-smokers. This might be partly driven by the higher GWG in smokers and, therefore, a higher insulin resistance (13, 22). The higher GWG observed for smokers could be linked to their unhealthy behaviors, including less frequent preventive screenings (10, 23–25) and the more frequent alcohol and recreational substance consumption observed in this study.

With regard to the combined effects of H1inP and smoking on birth weight, we only found three studies (20, 26, 27). The first study showed similar results in 400 Scandinavian women (26). The second study found in around 4,000 Finnish women that, in those without H1inP, the offspring birth weight was lowest in smokers, whereas in women with H1inP, the smoking status did not influence the offspring birth weight (20). The latter study did not explore the rate of LGA babies per se, and the changes in birth weight might have been driven by the different gestational ages at birth depending on the H1inP and smoking status. The third study, which included all Finnish primiparous women with singleton pregnancies between 2006 and 2018 (*n* = 290,602), found, as we did, that smoking and H1inP had opposing effects on fetal growth. Furthermore, compared with smoking after the first trimester of pregnancy, the cessation of smoking during the first trimester was associated with greater head circumference and birth weight in newborns (27).

In the present study, the rate of LGA babies in non-smokers without H1inP was similar to that in smokers with H1inP. However, we did not observe a lower rate of cesarean section or shoulder dystocia in smokers compared with non-smokers. Furthermore, the risk of severe SGA babies was increased in smokers regardless of the H1inP status, as previously reported (1, 28). This is likely due to several mechanisms (1–3), such as placental dysfunction through nicotine exposure (29), smoking-related altered endometrial maturation (30), and immune response and endothelial function (31).

### Other outcomes

In this study, smoking was positively associated with hypertensive disorders, including preeclampsia, but only in women with H1inP. A systematic review and meta-analysis of prospective studies reported a negative association between smoking during pregnancy and the risk of preeclampsia, even after adjustment for several confounders including diabetes (32). However, we did not find any study investigating the impact of the combined effect of H1inP and smoking on hypertensive disorders. Smoking and H1inP both increase placental hypoplasia with fetal vascular perfusion lesions (33), “two pathways” that increase hypoxia and oxidative stress that may converge on preeclampsia, and a worse neonatal condition (likely expressed in a high rate of NICU admissions). Previous studies have shown the separate impacts of smoking (34) and of H1inP, particularly when the glucose values are high at diagnosis (12), on malformations. Our results, although exploratory, suggest that the combination of both is associated with the highest prevalence of malformations. This should be investigated further.

### Strengths and limitations

A strength of this study is that it involved a large multi-ethnic cohort with prospective recruitment over a decade, allowing to explore the effects of smoking and H1inP and their combination on several adverse pregnancy outcomes, even if the event rates for neonatal hypoglycemia or stillbirth were low (35). We were also able to adjust for several cofounders, which also included the consumption of alcohol (36) and recreational substances (37).

The study also has several limitations. Firstly, smoking was self-reported. However, previous studies found a good validity of self-reported tobacco use when compared with measured plasma cotinine levels [31]. Secondly, we were unable to evaluate the impact of smoking at different gestational time points, and we had no quantitative data on cigarette smoking or a decrease in smoking quantity. In addition, despite the large cohort, the number of LGA babies in women with H1inP who smoked (10 out of 115) was relatively low. Moreover, we could not study placental lesions, whereas smoking-induced complications are likely driven by placental dysfunction (1–4). Finally, we had no data on paternal smoking and, thus, passive tobacco exposure (3).

### Perspectives

Our adjusted data suggest that further studies should examine the role of earlier or stricter glucose management in women with H1inP. Smoking is associated with many adverse pregnancy outcomes, to which life span consequences for the future infant, such as metabolic diseases, attention disorders, respiratory dysfunction, and even sudden death, should be added (1, 3). Based on the data from this study and on previous data, women who smoke during pregnancy should be targeted as they have a higher GWG compared with non-smokers and nevertheless have a high rate of severely growth-restricted babies, which may even be underestimated (38).

Finally, the results of this study argue for a particular attentive screening for hypertensive disorders in smokers with H1inP. As fetal growth may be normal in these women, they should particularly be monitored for blood pressure and placental function (e.g., by Doppler ultrasound, biomarkers, or fetal tolerance to late-term contractions) on the one hand and the quality of dietary observance and glycemic level on the other hand.

Further research should investigate the pathophysiological mechanisms related to the impact of smoking on insulin resistance, inflammation, and placental function in the presence of normal and increased glucose levels throughout pregnancy.

## Conclusion

Smoking and H1inP have opposing independent effects on fetal growth that therefore may appear normal in women with H1inP who smoke. Smoking among women with H1inP could mask the risk of maternal hyperglycemia for LGA babies. This might provide a false sense of security for women with H1inP who smoke, as it will hide a particular risk of hypertensive disorders during pregnancy and later severe SGA babies. These findings, together with the smoking- and H1inP-related life span consequences for both the child to be born and the mother, further argue for a timely smoking cessation in pregnant women.

## Data Availability

The raw data supporting the conclusions of this article will be made available by the authors, without undue reservation.
